# Diagnostic Utility of Measuring Cerebral Atrophy in the Behavioral Variant of Frontotemporal Dementia and Association With Clinical Deterioration

**DOI:** 10.1001/jamanetworkopen.2021.1290

**Published:** 2021-03-11

**Authors:** Ignacio Illán-Gala, Neus Falgàs, Adit Friedberg, Sheila Castro-Suárez, Ophir Keret, Nicole Rogers, Didem Oz, Salvatore Nigro, Andrea Quattrone, Aldo Quattrone, Amy Wolf, Kyan Younes, Miguel Santos-Santos, Sergi Borrego-Écija, Yann Cobigo, Oriol Dols-Icardo, Albert Lladó, Raquel Sánchez-Valle, Jordi Clarimon, Rafael Blesa, Daniel Alcolea, Juan Fortea, Alberto Lleó, Lea T. Grinberg, Salvatore Spina, Joel H. Kramer, Gil D. Rabinovici, Adam Boxer, Maria Luisa Gorno Tempini, Bruce L. Miller, William W. Seeley, Howard J. Rosen, David C. Perry

**Affiliations:** 1 Sant Pau Memory Unit, Department of Neurology, Hospital de la Santa Creu i Sant Pau, Biomedical Research Institute Sant Pau, Universitat Autònoma de Barcelona, Barcelona, Spain; 2 Atlantic Fellow for Equity in Brain Health, Department of Neurology, University of California, San Francisco; 3 Alzheimer’s Disease and Other Cognitive Disorders Unit, Department of Neurology, Hospital Clínic, Institut d’Investigació Biomèdica August Pi i Sunyer, University of Barcelona, Barcelona, Spain; 4 Neuroscience Centre, Magna Graecia University, Catanzaro, Italy; 5 Department of Medical and Surgical Sciences, Institute of Neurology, Magna Graecia University, Catanzaro, Italy; 6 Neuroimaging Research Unit, Institute of Molecular Bioimaging and Physiology, National Research Council, Catanzaro, Italy; 7 Memory and Aging Center, Department of Neurology, University of California, San Francisco

## Abstract

**Importance:**

The presence of atrophy on magnetic resonance imaging can support the diagnosis of the behavioral variant of frontotemporal dementia (bvFTD), but reproducible measurements are lacking.

**Objective:**

To assess the diagnostic and prognostic utility of 6 visual atrophy scales (VAS) and the Magnetic Resonance Parkinsonism Index (MRPI).

**Design, Setting, and Participants:**

In this diagnostic/prognostic study, data from 235 patients with bvFTD and 225 age- and magnetic resonance imaging–matched control individuals from 3 centers were collected from December 1, 1998, to September 30, 2019. One hundred twenty-one participants with bvFTD had high confidence of frontotemporal lobar degeneration (FTLD) (bvFTD-HC), and 19 had low confidence of FTLD (bvFTD-LC). Blinded clinicians applied 6 previously validated VAS, and the MRPI was calculated with a fully automated approach. Cortical thickness and subcortical volumes were also measured for comparison. Data were analyzed from February 1 to June 30, 2020.

**Main Outcomes and Measures:**

The main outcomes of this study were bvFTD-HC or a neuropathological diagnosis of 4-repeat (4R) tauopathy and the clinical deterioration rate (assessed by longitudinal measurements of Clinical Dementia Rating Sum of Boxes). Measures of cerebral atrophy included VAS scores, the bvFTD atrophy score (sum of VAS scores in orbitofrontal, anterior cingulate, anterior temporal, medial temporal lobe, and frontal insula regions), the MRPI, and other computerized quantifications of cortical and subcortical volumes. The areas under the receiver operating characteristic curve (AUROC) were calculated for the differentiation of participants with bvFTD-HC and bvFTD-LC and controls. Linear mixed models were used to evaluate the ability of atrophy measures to estimate longitudinal clinical deterioration.

**Results:**

Of the 460 included participants, 296 (64.3%) were men, and the mean (SD) age was 62.6 (11.4) years. The accuracy of the bvFTD atrophy score for the differentiation of bvFTD-HC from controls (AUROC, 0.930; 95% CI, 0.903-0.957) and bvFTD-HC from bvFTD-LC (AUROC, 0.880; 95% CI, 0.787-0.972) was comparable to computerized measures (AUROC, 0.973 [95% CI, 0.954-0.993] and 0.898 [95% CI, 0.834-0.962], respectively). The MRPI was increased in patients with bvFTD and underlying 4R tauopathies compared with other FTLD subtypes (14.1 [2.0] vs 11.2 [2.6] points; *P* < .001). Higher bvFTD atrophy scores were associated with faster clinical deterioration in bvFTD (1.86-point change in Clinical Dementia Rating Sum of Boxes score per bvFTD atrophy score increase per year; 95% CI, 0.99-2.73; *P* < .001).

**Conclusions and Relevance:**

Based on these study findings, in bvFTD, VAS increased the diagnostic certainty of underlying FTLD, and the MRPI showed potential for the detection of participants with underlying 4R tauopathies. These widely available measures of atrophy can also be useful to estimate longitudinal clinical deterioration.

## Key Points

**Question:**

Can widely available measures of atrophy on magnetic resonance imaging increase diagnostic certainty of underlying frontotemporal lobar degeneration (FTLD) and estimate clinical deterioration in the behavioral variant of frontotemporal dementia (bvFTD)?

**Findings:**

This diagnostic/prognostic study investigated the clinical utility of 5 validated visual atrophy scales (VAS) and the Magnetic Resonance Parkinsonism Index. When combined, VAS showed excellent diagnostic performance for differentiating between bvFTD with high and low confidence of FTLD and for the estimation of longitudinal clinical deterioration, whereas the Magnetic Resonance Parkinsonism Index was increased in bvFTD with underlying 4-repeat tauopathies.

**Meaning:**

These findings suggest that, in bvFTD, VAS can be used to increase diagnostic certainty of underlying FTLD and estimate longitudinal clinical deterioration.

## Teaser

This diagnostic/prognostic study assesses the utility of 6 visual atrophy scales and the Magnetic Resonance Parkinsonism Index in patients with behavioral variant frontotemporal dementia to distinguish those with high vs low confidence of frontotemporal lobar degeneration.

## Introduction

The behavioral variant of frontotemporal dementia (bvFTD) is the leading clinical presentation of frontotemporal lobar degeneration (FTLD).^1^ According to bvFTD diagnostic criteria, the presence of frontal or anterior temporal cerebral atrophy on magnetic resonance imaging (MRI) can be used to increase diagnostic certainty of underlying FTLD, and longitudinal studies have shown that cortical atrophy is associated with a faster clinical deterioration.^2,3,4^ However, objective and reproducible measurements of atrophy are lacking, and the specific value of MRI measures for differentiating between cases with nonneurodegenerative bvFTD and those with underlying FTLD is unclear.^5^ Previous studies applied sophisticated data-driven approaches to characterize atrophy, but these methods may be difficult to replicate across centers.^6,7^ On the contrary, visual atrophy scales (VAS) represent accessible and reliable measures of cerebral atrophy.^8^

An additional challenge is that bvFTD is associated with multiple FTLD subtypes, some of which are characterized by subcortical atrophy at diagnosis (eg, bvFTD with underlying progressive supranuclear palsy [PSP] or corticobasal degeneration [CBD]).^9^ The Magnetic Resonance Parkinsonism Index (MRPI) allows for the quantification of the relative volume loss in the midbrain and superior cerebellar peduncle and has shown excellent performance for the diagnosis of PSP, even before the emergence of canonical motor symptoms.^10^

In this multicenter study, we explored the clinical value of 6 VAS and the MRPI for differentiating between participants with bvFTD with high and low confidence of FTLD and healthy control individuals. We also examined the role of these accessible measures of atrophy for estimating underlying pathology and clinical deterioration rate.

## Methods

### Participant Selection

**Figure 1.**
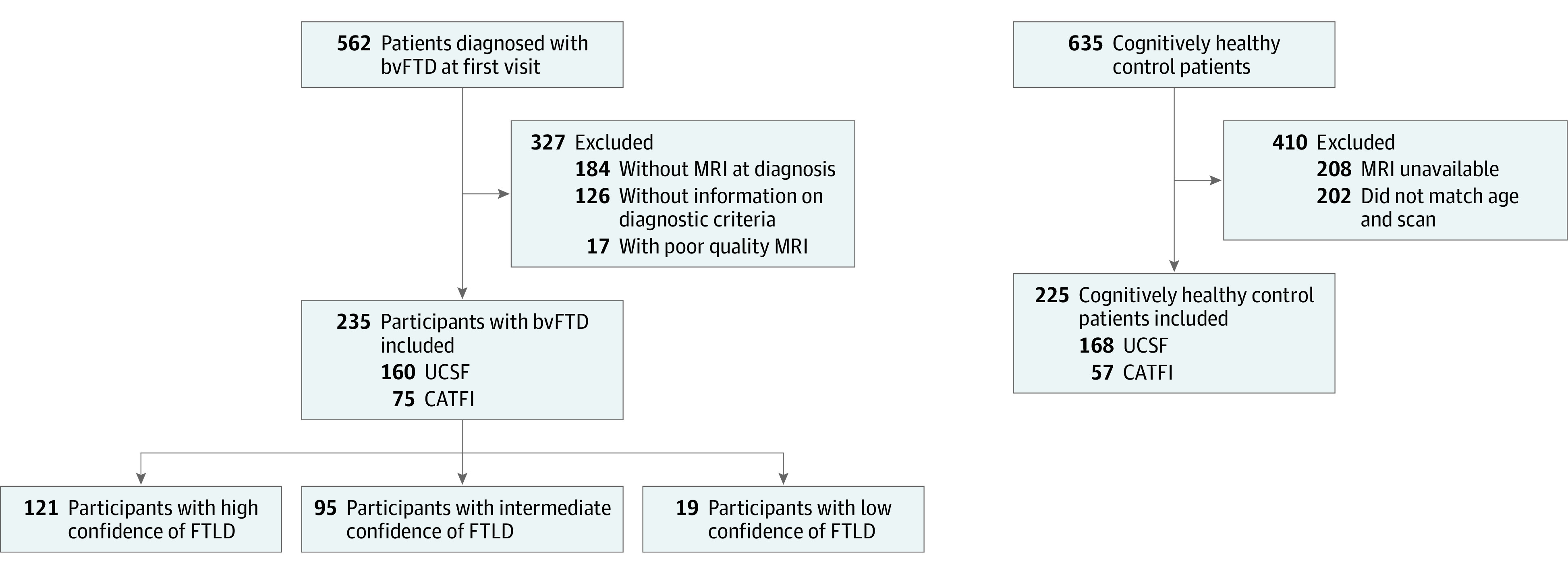
Flowchart of the Sample Composition bvFTD indicates behavioral variant of frontotemporal dementia; CATFI, Catalan Frontotemporal Dementia Initiative; FTLD, frontotemporal lobar degeneration; MRI, magnetic resonance imaging; and UCSF, University of California, San Francisco.

Figure 1 shows a flowchart of the sample composition for this diagnostic/prognostic study. Inclusion criteria for bvFTD participants were (1) meeting the International Behavioral Variant FTD Criteria Consortium revised guidelines for the diagnosis of at least possible bvFTD^2^ and (2) having MRI findings available for analysis at the time of diagnosis. Participants were recruited at 3 different centers: 160 at the University of California, San Francisco, Memory and Aging Center, 59 at Hospital de Sant Pau, Barcelona, Spain, and 16 at the Hospital Clinic, Barcelona, Spain. All patients underwent a complete clinical history, physical examination, neuropsychological evaluation, and structural brain imaging. A total of 225 age-matched healthy participants were also included as imaging controls. All controls had normal cognitive performance according to local normative data^11^ and did not have any neurological, psychiatric, or other major medical illnesses. This study followed the Strengthening the Reporting of Observational Studies in Epidemiology (STROBE) reporting guideline. The study was approved by the institutional review board of each center and was conducted following the Declaration of Helsinki. Written informed consent was obtained from all participants.

### Clinical Evaluation and Detailed Behavioral Assessment

Data were collected from December 1, 1998, through September 30, 2019. At presentation, the following measures of clinical deterioration were recorded: the Clinical Dementia Rating (CDR), the CDR Sum of Boxes (CDR-SOB),^12^ the Mini-Mental State Examination (MMSE),^13^ and the frequency-by-severity and scores of Neuropsychiatric Inventory.^14^ During follow-up of participants with bvFTD, the clinical features were reviewed, and we recorded additional measurements of MMSE (227 at baseline, 92 at year 1, and 45 at year 2) and CDR-SOB (199 at baseline, 86 at year 1, and 35 at year 2). The development of any additional syndromes, including amyotrophic lateral sclerosis, PSP–Richardson syndrome (PSP-RS), and semantic variant primary progressive aphasia, was also registered.^15,16,17,18^

### Classification of bvFTD Participants

Participants with bvFTD were classified in 3 groups based on the certainty of underlying FTLD. In the first group, we included participants with bvFTD with high confidence of FTLD (bvFTD-HC). This group was composed of 68 participants with autopsy-confirmed FTLD (including 24 FTLD mutations [15 *C9orf72*, 6 *GRN*, and 3 *MAPT*] and 7 with a second FTD-spectrum syndrome developed during follow-up [2 amyotrophic lateral sclerosis, 2 PSP-RS, and 3 semantic variant primary progressive aphasia]),^9,19^ 48 with FTLD-related mutations,^9,11^ and 36 who developed a second FTD-spectrum syndrome during follow-up (22 with amyotrophic lateral sclerosis,^15^ 10 with PSP-RS,^16^ and 4 with semantic variant primary progressive aphasia^17^). In the second group, we included 19 participants with bvFTD with low confidence of FTLD (bvFTD-LC). This group included 13 participants who received an alternative, non–FTD-spectrum syndromic diagnosis during follow-up and 6 in whom FTLD was ruled out on autopsy results (eTable 1 in the Supplement). Of note, the bvFTD-LC group included participants verifying bvFTD criteria at diagnosis but without clinical deterioration or alternative psychiatric diagnosis during follow-up (bvFTD phenocopies). Of note, all participants included in the bvFTD-LC group had negative test results for the *C9orf72* expansion. In the third group, we included the remainder of the participants with bvFTD with intermediate confidence of FTLD (bvFTD-IC) (n = 95).

### Structural MRI Acquisition

The images were acquired on scanners from 7 different manufacturers using different imaging protocols (eTable 2 in the Supplement). Magnetic field strength varied at 1.5 T (48 scans), 3.0 T (385 scans), and 4.0 T (27 scans).

### Rating of Cerebral Atrophy With VAS

Six neurologists (I.I.-G., N.F., A.F., S.C.-S., O.K., and N.R.) blinded to any clinical information applied 6 VAS in all participants. These previously published VAS were optimized to improve their usability and consistency (eg, precise slice selection for rating), validated in a large postmortem study by Harper et al,^8^ and included a 5-point anterior temporal scale,^20,21^ a 5-point medial temporal lobe atrophy scale,^22^ a 4-point posterior atrophy scale,^23^ a 4-point orbitofrontal scale,^8^ a 4-point anterior cingulate scale,^8^ and a 4-point frontoinsula scale.^8^ The application of these VAS takes a mean of less than 3 minutes per participant.^8^ All raters first received a 1-hour training session. After this training session, all raters applied the scale in an independent data set of 20 MRI scans (1 measurement for each hemisphere, 40 measurements for each scale). Each evaluator received feedback on their performance compared with other raters in this first training data set, and consistent disagreement for each scale was discussed in a second meeting. Before the beginning of the study, all raters assessed a second set of 20 participants included in the study to provide additional independent validation of the results. We confirmed the reliability of VAS measurements in an independent data set (eTable 3 in the Supplement). Images were rated in native space, in keeping with standard clinical reads, and separate scores were recorded for regions in the left and right hemispheres. Also, to aid rating consistency, all the raters were trained with the same instructions, slice selection, and reference images as in the validation by Harper and collaborators.^8^ Left and right hemisphere scores were added to provide a single measure of atrophy for each of the 6 regions (eTable 4 in the Supplement). We also aimed to obtain a single measure of frontotemporal atrophy that could be easily calculated by clinicians at the bedside to identify patients with bvFTD-HC: the bvFTD atrophy score. To achieve this, we added the scores of 5 of the 6 VAS included in this study (anterior cingulate, orbitofrontal, frontal insula, anterior temporal, and medial temporal lobe). The bvFTD atrophy score can range from 0 to a maximum of 44 (22 per hemisphere), with higher scores indicating greater levels of atrophy. Of note, the selection of these VAS for the calculation of the bvFTD atrophy score was based in their ability to discriminate bvFTD with underlying FTLD from controls and other dementias in a previous pathology-proven study.^8^

### Cortical Thickness and Subcortical Grey Matter Volumes

The MRIs were processed with the CAT12 toolbox within SPM12 (running in MATLAB r2019b; MathWorks) to obtain the mean cortical thickness in each region on the Desikan atlas and subcortical gray matter volumes in the neuromorphometrics atlas.^24,25^ We then calculated the mean cortical thickness at each region in the Desikan atlas and the volumes of subcortical gray matter structures in the neuromorphometrics atlas, as implemented in CAT12. We also calculated the MRPI, which is derived from midbrain and pons areas and middle and superior cerebellar peduncle widths following a previously validated, fully automated method.^26,27^

### Measures of Atrophy for the Diagnosis of bvFTD

We hypothesized that the bvFTD atrophy score would provide similar diagnostic accuracy for the identification of bvFTD-HC to measures obtained by more sophisticated data-driven approaches. We were particularly interested in comparing the diagnostic accuracy of the different atrophy measures for the differentiation between bvFTD-HC and controls and between bvFTD-HC and bvFTD-LC. We designed 2 different discriminant factor analyses (DFA; Wilks lambda and stepwise selection of independents method) for the identification of bvFTD-HC in each of these 2 subsamples. We performed additional DFA in other subsamples for other secondary analyses, including participants classified in the bvFTD-IC group (eTable 5 in the Supplement). We then calculated the areas under the receiver operating characteristic curve (AUROC) for all measures (atrophy measures and estimated probabilities obtained from DFA models) in each subsample. We performed a secondary analysis to explore whether MRPI could detect participants with bvFTD with high risk of underlying PSP pathology by calculating the AUROC of MRPI to differentiate between participants with bvFTD progressing to PSP-RS or pathology proven PSP-CBD and other FTLD cases. Finally, to determine whether the atrophy measures were significantly different from each other, we compared receiver operating curves with a nonparametric test that accounts for the correlation of the curves.^28^ This test takes advantage of the equality between the Mann-Whitney statistic for comparing distributions and the AUROC when computed by the trapezoidal rule.

### Measures of Atrophy for the Estimation of Clinical Deterioration

We aimed to compare the ability of the bvFTD atrophy score to estimate clinical deterioration with other automated measures that capture FTLD-related atrophy at the single subject level. For doing this, we first calculated the mean cortical thickness and subcortical gray matter volume for both hemispheres for all the analyses, as in previous bvFTD studies (eTable 4 in the Supplement).^7^ Then, we obtained the mean frontotemporal cortical thickness by calculating the mean cortical thickness of all the frontal and temporal regions in the Desikan atlas. Because subcortical gray matter volumes (but not cortical thickness) depend on total intracranial volume,^29^ we divided subcortical gray matter volumes by total intracranial volume of each participant to obtain normalized subcortical volumes.

### Comparison of Imaging Methods for Capturing Atrophy

To illustrate the ability of the bvFTD atrophy score to capture frontotemporal atrophy in participants with bvFTD, we studied the correlation of cortical thickness with the bvFTD atrophy score in all participants with bvFTD using multiple regressions with individual bvFTD atrophy scores as the variable of interest and age, sex, and MRI scanner as covariates. We considered a significant statistical threshold of 2-sided *P* < .05, corrected for false discovery rate, using an extent threshold of the expected vertices per cluster. Finally, we compared the bvFTD-LC group (n = 19) and subgroups (ie, no FTLD [n = 6] and psychiatric subgroups [n = 13]) with healthy controls following the same approach. In these analyses, we set a less restrictive threshold for statistical significance of 2-sided *P* < .001 to increase our sensitivity to capture small to moderate effect sizes owing to the relatively small sample size of the bvFTD-LC group.

### Clinical Deterioration Analyses of Participants With bvFTD 

In longitudinal studies, linear mixed-effects have proven to be powerful tools for identifying variables where baseline values are associated with different rates of changes in clinical deterioration.^30^ We fitted linear mixed-effects analyses controlling for age, sex, genetic status (presence or absence of an FTLD-related mutation), and different measures of atrophy (bvFTD atrophy score, MRPI, frontotemporal cortical thickness, and subcortical gray matter volume) to estimate clinical deterioration over time in participants with bvFTD, as measured by CDR-SOB. All models included a random patient-specific intercept and a random patient-specific slope. These random effects account for patient heterogeneity in baseline CDR-SOB and its rate of increase that is not explained by the predictive factors in the model. A term for biomarker by time interaction was used to study the association between the baseline biomarker level and CDR-SOB over time. As a secondary analysis, we fitted additional linear mixed-effects models replacing CDR-SOB with MMSE (as an alternative measure of general clinical deterioration in bvFTD).^31^ As in similar previous studies,^32^ all linear mixed models were designed with a compound symmetry covariance structure (owing to the relative homogeneity in the covariance of effects). Of note, we obtained essentially the same results when linear mixed models were fitted with an unstructured covariance.

### Other Statistical Analysis

Data were analyzed from February 1 to June 30, 2020. Data were explored for normality using the Kolmogorov-Smirnov test. Between-group differences in baseline characteristics and measures of atrophy were assessed using the 2-tailed unpaired *t* test, analysis of variance, Mann-Whitney test, or Kruskal-Wallis test for continuous variables and the χ^2^ test for categorical data. We also performed secondary analyses to compare MRPI levels among pathology-proven PSP-CBD, cases progressing to PSP-RS, and other cases with FTLD. We applied the Spearman correlation index (ρ value) to study the correlation between measures of clinical deterioration and measures of cerebral atrophy with bootstrapping-based 95% CIs (bias corrected and accelerated for 1000 samples). Statistical significance for all tests was set at 5% (α = .05), and all statistical tests were 2 sided. All analyses were performed using SPSS, version 25 (IBM Corp).

## Results

### Demographic and Clinical Characteristics of Participants

Among the 460 included participants (296 men [64.3%] and 164 women [35.7%]; mean [SD] age, 62.6 [11.4] years), age at MRI (mean [SD], 63.3 [10] vs 61.8 [12.6] years) and educational level (mean [SD], 14.7 [4.4] vs 15.3 [3.8] years) were similar in the bvFTD and control groups (Table). Mean (SD) age at symptom onset (range, 56.2 [10.3] to 59.5 [10.0] years), MMSE score (range, 22.7 [6.7] to 26.7 [2.6] years), and CDR-SOB score (range, 4.6 [1.9] to 7.4 [3.3]) were similar among bvFTD subgroups, but mean (SD) follow-up time was higher in the bvFTD-LC group (2.3 [1.3] years) than in the bvFTD-IC (1.2 [1.5] years) and bvFTD-HC (1.2 [1.3] years) groups. As shown in eFigure 1 in the Supplement, the behavioral profile of bvFTD subgroups was similar, but the bvFTD-LC group had higher scores in the Neuropsychiatric Inventory irritability domain (mean [SD], 6.3 [4.6]) than the bvFTD-IC (mean [SD], 3.3 [4.3]) and bvFTD-HC (mean [SD], 2.3 [3.1]) groups (*P* = .003).

**Table.** Demographic and Clinical Characteristics and Measures of Cerebral Atrophy

Characteristic	Participant group^a^				
	bvFTD-IC (n = 95)	bvFTD-HC (n = 121)	bvFTD-LC (n = 19)	All bvFTD (n = 235)	Control (n = 225)
Age at symptom onset, y	59.5 (10.0)	56.7 (10.8)	56.2 (10.3)	57.7 (10.5)	NA
Age at MRI, y	64.9 (9.6)	62.1 (10.4)	63.4 (10.1)	63.3 (10)	61.8 (12.6)
No. (%) male	63 (66.3)	86 (71.1)	16 (84.2)	165 (70.2)	131 (58.2)^b^
Educational level, y	15.0 (4.2)	14.7 (4.4)	13.1 (4.9)	14.7 (4.4)	15.3 (3.8)
MMSE score^c^	23.5 (6.6)	22.7 (6.7)	26.7 (2.6)	23.4 (6.5)	29.0 (1.1)^b^^,^^d^
CDR-SOB score^e^	5.8 (3.4)	7.4 (3.3)	4.6 (1.9)	6.5 (3.4)	0 (0.1)^b^^,^^d^
Time of follow-up, y	1.2 (1.5)	1.2 (1.3)	2.3 (1.3)^f^	1.3 (1.4)	NA
Cohort, No. CATFI/UCSF	28/67	39/82	8/11	75/160	57/168
VAS score					
Orbitofrontal	2.9 (2.0)	3.0 (2.0)	0.8 (1.2)^f^	2.8 (2.0)	0.8 (1.1)^b^^,^^g^
Anterior cingulate	3.7 (1.7)	4.2 (1.6)	1.9 (1.8)^f^	3.8 (1.8)	1.6 (1.4)^b^^,^^g^
Anterior temporal	2.9 (1.5)	3.2 (1.6)	1.7 (0.9)^f^	3.0 (1.6)	1.6 (1.0)^b^^,^^g^
Medial temporal lobe	2.9 (2.3)	3.2 (2.1)	1.3 (1.9)^f^	2.9 (2.2)	0.6 (1.2)^b^^,^^g^
Frontal insula	3.1 (1.7)	3.4 (1.7)	1.0 (1.4)^f^	3.1 (1.8)	1.2 (1.2)^b^^,^^g^
Posterior atrophy	0.6 (1.0)	0.9 (1.1)	0.4 (0.7)	0.8 (1.1)	0.7 (1.0)
bvFTD atrophy^h^	15.7 (7.2)	16.9 (6.4)	6.7 (5.8)^f^	15.6 (7.2)	5.8 (4.0)^b^^,^^g^
MRPI					
Midbrain volume, mm^3^	106.2 (20.4)	104.3 (21.1)	125.6 (20.2)^f^	106.8 (21.4)	128.0 (23.0)^b^^,^^g^
Pons volume, mm^3^	490.3 (57.7)	493.6 (60.0)	519.0 (54.9)	494.3 (54.4)	494.3 (52.7)
Superior cerebellar peduncle width, mm	3.8 (0.4)	3.8 (0.4)	3.7 (0.5)	3.8 (0.4)	3.9 (0.4)^b^
Middle cerebellar peduncle width, mm	8.8 (0.8)	8.9 (0.8)	9.1 (0.9)	8.9 (0.8)	9.1 (0.7)^b^^,^^i^
Midbrain to pons ratio	0.22 (0.04)	0.21 (0.04)	0.24 (0.04)	0.22 (0.04)	0.26 (0.05)
Score	11.3 (2.7)	11.6 (2.7)	10.4 (2.3)	11.4 (2.7)	9.4 (2.2)^b^^,^^g^
Cortical thickness and subcortical gray matter measures					
Frontotemporal cortical thickness, mm^j^	2.6 (0.2)	2.5 (0.2)	2.8 (0.2)^f^	2.6 (0.2)	2.9 (0.1)^b^^,^^f^
Subcortical gray matter ratio^k^	1.3 (0.2)	1.2 (0.2)	1.4 (0.2)^f^	1.3 (0.2)	1.6 (0.2)^b^^,^^d^

Abbreviations: bvFTD, behavioral variant of frontotemporal dementia; CATFI, Catalan Frontotemporal Dementia Initiative; CDR-SOB, Clinical Dementia Rating Sum of Boxes; HC, high confidence; IC, intermediate confidence; LC, low confidence; MMSE, Mini-Mental State Examination; MRPI, Magnetic Resonance Parkinsonism Index; NA, not applicable; UCSF, University of California, San Francisco; VAS, visual atrophy scales.

^a^ Unless otherwise indicated, data are expressed as mean (SD).

^b^ *P* < .05 compared with all-bvFTD group.

^c^ Scores range from 0 to 30, with higher scores indicating better cognition.

^d^ *P* < .05 compared with bvFTD-IC, bvFTD-HC, and bvFTD-LC groups.

^e^ Scores range from 0 to 18, with higher scores indicating more advanced dementia. This measure was available for 199 participants with bvFTD (84.7%) and 188 healthy controls (83.6%).

^f^ *P* < .05 compared with bvFTD-IC and bvFTD-HC.

^g^ *P* < .05 compared with bvFTD-HC and bvFTD-LC.

^h^ The bvFTD atrophy score ranges from 0 to 34, with higher scores indicating more cortical atrophy, as measured with VAS. The bvFTD atrophy score results from the addition of orbitofrontal, anterior cingulate, anterior temporal, medial temporal lobe, and frontal insula atrophy scores of both hemispheres.

^i^ *P* < .05 compared with bvFTD-IC.

^j^ Indicates mean of cortical thickness at frontal and temporal regions of both hemispheres.

^k^ Indicates mean of volumes of accumbens, amygdala, caudate nucleus, hippocampus, putamen, and thalamus of both hemispheres divided by the resulting volume by total intracranial volume.

### Group Differences in Cerebral Measurements of Atrophy

**Figure 2.**
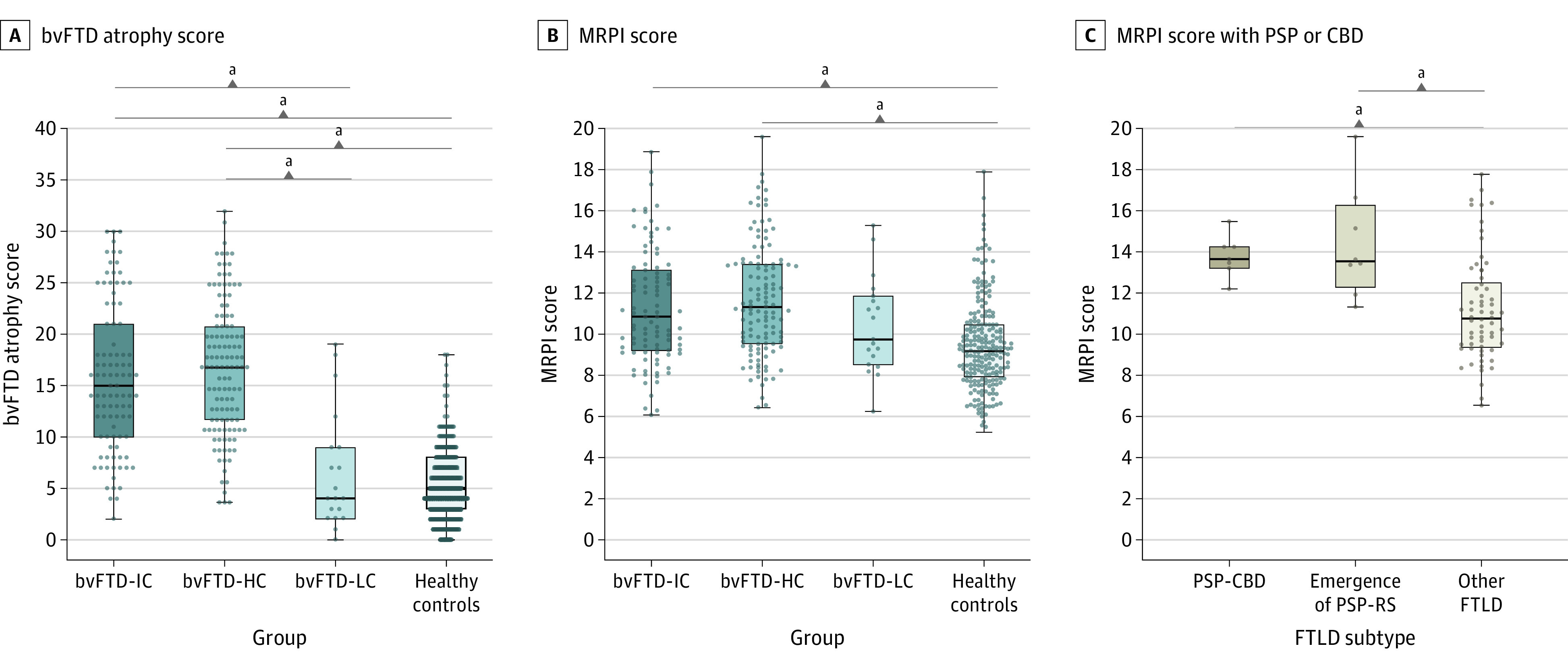
Group Comparison of Behavioral Variant of Frontotemporal Dementia (bvFTD) Atrophy Score and Magnetic Resonance Parkinsonism Index (MRPI) Score Atrophy scores and MRPI scores are compared among participants with intermediate (bvFTD-IC), high (bvFTD-HC), and low (bvFTD-LC) confidence of frontotemporal lobar degeneration and healthy controls. The MRPI scores are compared in participants with bvFTD with progressive supranuclear palsy or corticobasal degeneration (PSP-CBD) or with emergence of PSP–Richardson syndrome (PSP-RS) during follow-up and in cases with remaining pathology-proven (other) FTLD. Atrophy scores range from 0 to 34, with higher scores indicating more cortical atrophy. MRPI scores range from 0 to 20, with higher scores indicating more midbrain superior peduncle atrophy. Horizontal lines indicate medians; boxes, quartile 1 to quartile 3; whiskers, minimum to maximum values; and dots, individual participant values. ^a^*P* < .05, adjusted for multiple comparisons (Bonferroni).

Among the bvFTD-IC and bvFTD-HC groups, the mean (SD) VAS scores in orbitofrontal (2.9 [2.0] and 3.0 [2.0], respectively), anterior cingulate (3.7 [1.7] and 4.2 [1.6], respectively), medial temporal lobe (2.9 [1.5] and 3.2 [1.6], respectively), and the frontal insula (3.1 [1.7] and 3.4 [1.7], respectively) regions and the total bvFTD atrophy score (15.7 [7.2] and 16.9 [6.4], respectively) were higher compared with bvFTD-LC (orbitofrontal, 0.8 [1.2]; anterior cingulate, 1.9 [1.8]; medial temporal, 1.3 [1.9]; frontal insula, 1.0 [1.4]; and total atrophy, 6.7 [5.8]) and control (orbitofrontal, 0.8 [1.1]; anterior cingulate, 1.67 [1.4]; medial temporal, 06 [1.2]; frontal insula, 1.2 [1.2]; and total atrophy, 5.8 [4.0]) groups (Table and Figure 2A). Of note, the bvFTD atrophy score showed an excellent correlation with cortical thickness in frontotemporal regions (eFigure 2 in the Supplement). Mean (SD) frontotemporal cortical thickness and subcortical gray matter volume were also decreased in the bvFTD-IC (2.6 [0.2] mm and 1.3 [0.2] mm^3^, respectively) and bvFTD-HC (2.5 [-0.2] mm and 1.2 [0.2] mm^3^, respectively) groups when compared with both the bvFTD-LC (frontotemporal cortical thickness, 2.8 [0.2] mm; subcortical gray matter volume, 1.4 [0.2] mm^3^) and control (frontotemporal cortical thickness, 2.9 [0.1] mm; subcortical gray matter volume, 1.6 [0.2] mm^3^) groups (Table). Regarding MRPI measures, mean (SD) midbrain volume was the sole metric reduced in bvFTD-IC (106.2 [20.4] mm^3^) and bvFTD-HC (104.3 [21.1] mm^3^) groups compared with bvFTD-LC (125.6 [20.2] mm^3^) and controls (128.0 [23.0] mm^3^) (Table). The mean (SD) MRPI score was increased in the bvFTD-IC (11.3 [2.7]) and bvFTD-HC (11.6 [2.7]) groups compared with controls (9.4 [2.2]) (Table and Figure 2B).

### Cerebral Measurements of Atrophy in FTLD Subgroups

ThenVAS, frontotemporal cortical thickness, and subcortical gray matter volume were similar between FTLD subtypes (eTable 6 in the Supplement). However, participants with bvFTD and underlying PSP or CBD and those without neuropathological confirmation who developed PSP-RS during follow-up (n = 8) had increased values of MRPI when compared with other pathologically proven FTLD cases (mean [SD], 14.1 [2.0] vs 11.2 [2.6]; *P* < .001) (Figure 2C). The diagnostic accuracy of MRPI to differentiate between pathology-proven PSP-CBD or cases progressing to PSP-RS and other FTLD cases was moderate (AUROC, 0.829; 95% CI, 0.739-0.919). Of note, the MRPI scores of bvFTD participants with pathology-proven PSP (n = 2) and CBD (n = 5) were similar (mean [SD], 14.5 [1.4] vs 13.5 [0.9]; *P* = .57).

### Diagnostic Accuracy of Atrophy Scales

**Figure 3.**
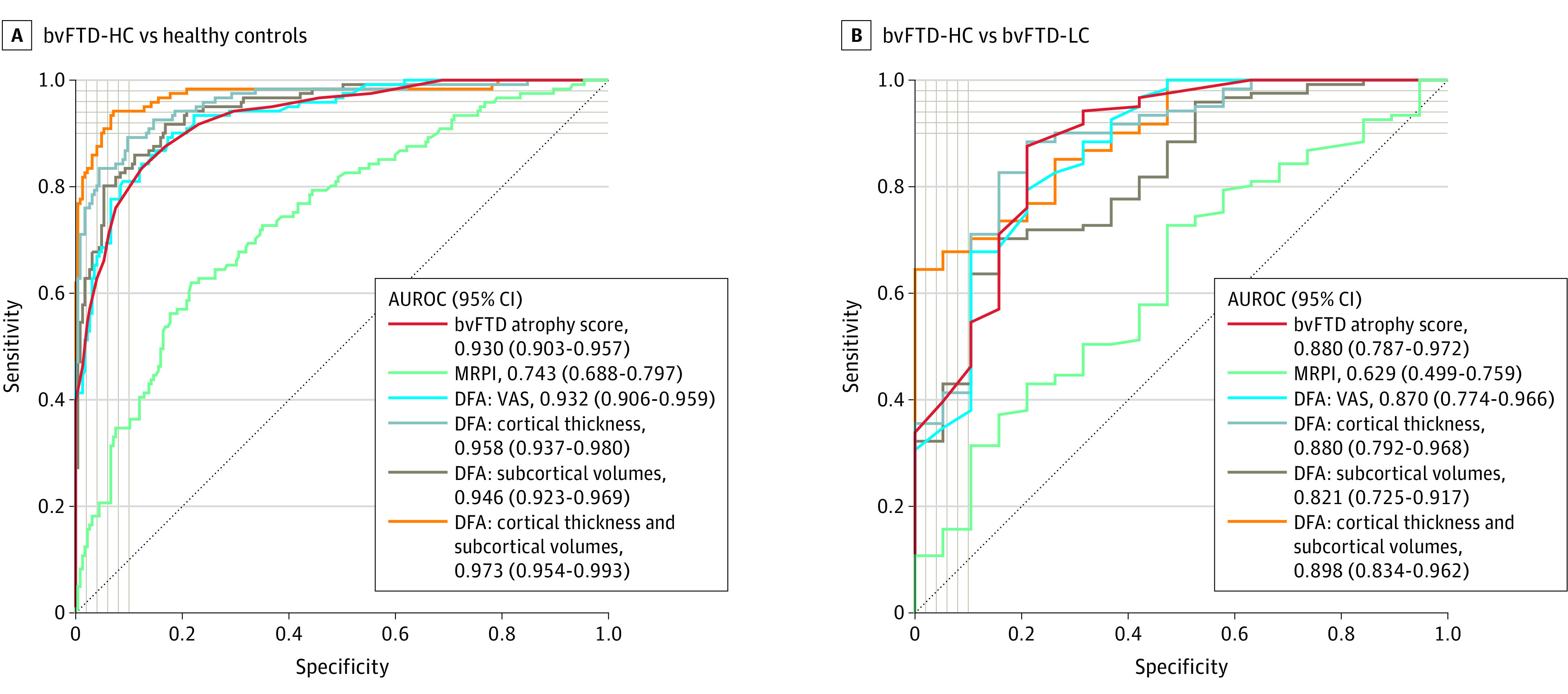
Diagnostic Accuracy A, Area under the receiver operating characteristic curves (AUROC) for the differentiation between participants with behavioral variant of frontotemporal dementia with high confidence (bvFTD-HC) of frontotemporal lobar degeneration (FTLD) (n = 121) and controls (n = 225). B, AUROC for the differentiation between participants with bvFTD-HC and those with bvFTD with low confidence of FTLD (bvFTD-LC) (n = 19). DFA indicates discriminant factor analyses; MRPI, Magnetic Resonance Parkinsonism Index; and VAS, visual atrophy scales.

Figure 3A shows the diagnostic accuracy of different atrophy measurements for the differentiation between bvFTD-HC and controls. Details on proposed cutoffs for the diagnosis of bvFTD with the bvFTD atrophy score can be found in eTable 7 in the Supplement. The bvFTD atrophy score showed an excellent diagnostic performance (AUROC, 0.930; 95% CI, 0.903-0.957), only outperformed by the DFA model combining cortical thickness and subcortical volume measures (AUROC, 0.973 [95% CI, 0.954-0.993]; *P* < .001). Importantly, the diagnostic accuracy of the bvFTD atrophy score was similar to DFA models that included VAS scores (AUROC, 0.932 [95% CI, 0.906-0.959]; *P* = .97), cortical thickness measures (AUROC, 0.958 [95% CI, 0.937-0.980]; *P* = .12), and subcortical volumes (AUROC, 0.946 [95% CI, 0.923-0.969]; *P* = .26) and was superior to MRPI (AUROC, 0.743 [95% CI, 0.688-0.797]; *P* < .001). We obtained essentially the same results when distinguishing a combined bvFTD-HC and bvFTD-IC group from controls (eFigure 3 in the Supplement) or when we restricted the analyses to the subgroup of bvFTD-HC with lower clinical severity (eFigure 4 in the Supplement). Moreover, the bvFTD atrophy score showed the second highest AUROC for the differentiation between bvFTD-HC and bvFTD-LC (0.880; 95% CI, 0.787-0.972) (Figure 3B). In this clinical scenario, the bvFTD atrophy score was similar to DFA models including VAS (AUROC, 0.870 [95% CI, 0.774-0.966]; *P* = .64), cortical thickness measures (AUROC, 0.880 [95% CI, 0.792-0.968]; *P* = .99), subcortical volumes (AUROC, 0.821 [95% CI, 0.725-0.917]; *P* = .06), and cortical thickness and subcortical volumes (AUROC, 0.898 [95% CI, 0.834-0.962]; *P* = .62).

### Estimation of Longitudinal Clinical Deterioration

**Figure 4.**
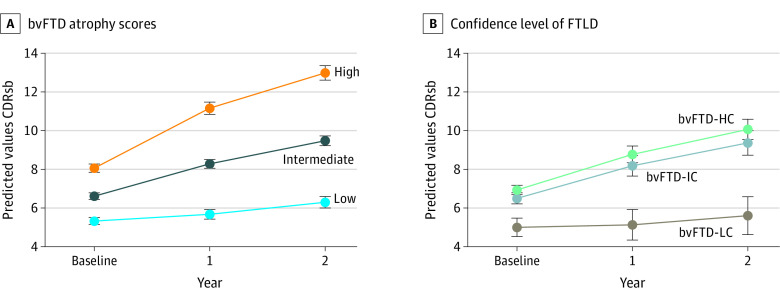
Association of Behavioral Variant of Frontotemporal Dementia (bvFTD) Atrophy Score With Progression Rate A, Estimated Clinical Dementia Rating Sum of Boxes (CDR-SOB) values from the linear mixed-effects model for low, intermediate, and high bvFTD visual atrophy scale scores. B, Estimated CDR-SOB values from the linear mixed-effects model for intermediate (bvFTD-IC), high (bvFTD-HC), and low (bvFTD-LC) confidence of frontotemporal lobar dementia (FTLD). Error bars indicate 95% CI.

Baseline bvFTD atrophy score was associated with an increased rate of clinical deterioration, as measured with both MMSE (change per atrophy score increase per year, 1.86 [95% CI, 0.99-2.73] points; *P* < .001) and CDR-SOB (change per atrophy score increase per year, 1.86 [95% CI, 0.99-2.73] points; *P* < .001) (Figure 4A and eTables 8 and 9 in the Supplement). Similar performance for the estimation of longitudinal increase in CDR-SOB was found for frontotemporal cortical thickness (change per atrophy score increase per year, 1.45 [95% CI, 0.60-2.31] points; *P* = .001) and subcortical gray matter volume (change per atrophy score point increase per year, 1.56 [95% CI, 0.55-2.56] points; *P* < .001). However, the MRPI was not associated with longitudinal clinical deterioration in participants with bvFTD. Finally, we compared clinical deterioration between bvFTD subgroups. As shown in Figure 4B, the bvFTD-LC group showed a slower clinical deterioration and had milder atrophy than bvFTD-IC and bvFTD-HC groups (eFigure 5 in the Supplement).

## Discussion

In this multicenter diagnostic/prognostic study, we found that a simple combination of 5 VAS (the bvFTD atrophy score) had good diagnostic accuracy for the identification of bvFTD caused by FTLD. Of note, the bvFTD atrophy score provided similar diagnostic accuracy to automated measures of cerebral atrophy and estimated progression in bvFTD. Another novel finding of this study is that the MRPI, a biomarker of midbrain atrophy, is increased in bvFTD with autopsy-confirmed PSP or CBD.

Current diagnostic criteria include the presence of “frontal and/or anterior temporal atrophy on MRI” to increase the diagnostic confidence of FTLD in bvFTD,^5^ but some cases may present with equivocal patterns of atrophy, and specific cutoffs for the definition of significant atrophy are lacking. Previous work showed low sensitivity of expert radiologists for the identification of frontotemporal atrophy,^33^ and other reports suggested a significant overlap in the atrophy between some cases with bvFTD and controls.^34^ It could be argued that clinicians not blinded to clinical information may have had increased sensitivity for the detection of participants with bvFTD. However, because we also showed that cases with bvFTD-HC and bvFTD-LC had a similar clinical presentation, relying on the subjective judgment of clinicians may also decrease the specificity of bvFTD diagnosis. Overall, our findings support the use of VAS as reliable and reproducible tools to increase the diagnostic confidence of FTLD in patients meeting diagnostic criteria for bvFTD at the first clinical encounter. Notwithstanding, several other automated morphometric MRI analyses (eg, machine learning algorithms) have also shown potential as diagnostic biomarkers in bvFTD, but further work is needed before these methods can be recommended for clinical use.^35^

A key finding of our study is that measures of cortical atrophy allowed accurate estimations of the clinical deterioration rate in bvFTD. Although previous studies have investigated the association between atrophy and clinical deterioration in bvFTD, these included a relatively small number of cases and did not use reproducible measures of atrophy.^3^ Interestingly, other studies have described the existence of different bvFTD subtypes, including a slowly progressive variant.^3,4^ Our results support that bvFTD with less cortical atrophy at diagnosis may also show a slower progression rate.^4,36,37^ In other previous longitudinal studies, participants with bvFTD and less atrophy also included bvFTD mimics (termed *phenocopies*) characterized by the absence of clinical deterioration over time and alternative psychiatric diagnosis.^38^ Our results support the view that many of these bvFTD mimics could be identified at the first clinical encounter with reproducible measures of cerebral atrophy. Additional studies are needed to investigate the role of other novel promising neuroimaging or fluid biomarkers such as cortical mean diffusivity^39^ or neurofilament light chain levels in plasma^40^ to increase the diagnostic accuracy of VAS and to differentiate bvFTD cases without underlying FTLD.

Another novel finding of our study is the increased values of MRPI in bvFTD participants with PSP or CBD on autopsy or developing PSP-RS during follow-up. Of note, diagnostic criteria for both PSP and CBD have been updated to include a frontal/cognitive behavioral or a frontospatial variant overlapping with the bvFTD syndrome.^41,42^ Our findings support the notion that bvFTD with underlying PSP or CBD could also be diagnosed before the emergence of canonical motor symptoms and signs supportive of PSP.^43^ This would be of utmost importance for the recruitment of participants with bvFTD for clinical trials targeting 4R tauopathies. Supporting our findings, another study^10^ reported that MRPI is also increased in patients who present with parkinsonism before the emergence of supranuclear palsy or postural instability. In our study, the diagnostic accuracy of MRPI alone for the identification of participants with PSP, CBD, or bvFTD developing PSP syndrome during follow-up was only moderate (AUROC, 0.829). Although these findings are encouraging, this observation is based on a relatively small number of participants, and larger pathologically proven studies are needed to precisely determine the diagnostic value of MRPI (alone or in combination with other biomarkers) for the differentiation of PSP and CBD pathology in bvFTD.

### Limitations

This study has some limitations. First, the bvFTD-LC group was small and included participants without autopsy confirmation. Despite this limitation, our results suggest that VAS and other measures of atrophy could be helpful to discriminate between bvFTD-LC participants and bvFTD-HC. This result is encouraging and deserves further investigation. Second, VAS included in this study did not assess subcortical cortical regions that could be relevant for the diagnosis of bvFTD (ie, thalamus or basal ganglia). Finally, we could not assess the exact precision of MRPI to detect the emergence of PSP- and CBD-related symptoms outside of a bvFTD presentation because these participants were not prospectively recruited in all centers.

## Conclusions

This diagnostic/prognostic study found that in bvFTD, VAS increased the diagnostic certainty of underlying FTLD, and the MRPI showed potential for the detection of participants with underlying 4R tauopathies. These widely available measures of atrophy can also be useful to estimate longitudinal clinical deterioration.

## Notes

Supplement. **eTable 1.** Clinical Characteristics of Participants Classified in bvFTD-LC Group **eTable 2.** Structural T1-Weighted Image Acquisition Protocols by Center **eTable 3.** Interrater Reliability Analyses of Visual Atrophy Scales **eTable 4.** AUROC for the Differentiation Between bvFTD-HC and Healthy Controls **eTable 5.** Diagnostic Accuracy for Each Discriminant Factor Analysis **eTable 6.** Measures of Cerebral Atrophy by FTLD Subtypes **eTable 7.** bvFTD Atrophy Score Cutoffs **eTable 8.** Linear Mixed-Effects Models for the Estimation of CDR-SOB Change **eTable 9.** Linear Mixed-Effects Models for the Estimation of MMSE Change **eFigure 1.** Clinical Characteristics of bvFTD Participants **eFigure 2.** Correlation Between bvFTD Atrophy Score and Cortical Thickness **eFigure 3.** Diagnostic Accuracy of Measures of Atrophy **eFigure 4.** Diagnostic Accuracy of Measures of Atrophy in the Subgroup of bvFTD-HC With Lower CDR-SOB **eFigure 5.** Cortical Thickness in bvFTD-LC
