# Supplementary material for: Diagnostic Utility of Measuring Cerebral Atrophy in the Behavioral Variant of Frontotemporal Dementia and Association With Clinical Deterioration
Source: JAMA Netw Open. 2021 Mar 11;4(3):e211290. doi: 10.1001/jamanetworkopen.2021.1290 (PMC7953307; doi:10.1001/jamanetworkopen.2021.1290)
Supplement: Supplement. — eTable 1. Clinical Characteristics of Participants Classified in bvFTD-LC Group eTable 2. Structural T1-Weighted Image Acquisition Protocols by Center eTable 3. Interrater Reliability Analyses of Visual Atrophy Scales eTable 4. AUROC for the Differentiation Between bvFTD-HC and Healthy Controls eTable 5. Diagnostic Accuracy for Each Discriminant Factor Analysis eTable 6. Measures of Cerebral Atrophy by FTLD Subtypes eTable 7. bvFTD Atrophy Score Cutoffs eTable 8. Linear Mixed-Effects Models for the Estimation of CDR-SOB Change eTable 9. Linear Mixed-Effects Models for the Estimation of MMSE Change eFigure 1. Clinical Characteristics of bvFTD Participants eFigure 2. Correlation Between bvFTD Atrophy Score and Cortical Thickness eFigure 3. Diagnostic Accuracy of Measures of Atrophy eFigure 4. Diagnostic Accuracy of Measures of Atrophy in the Subgroup of bvFTD-HC With Lower CDR-SOB eFigure 5. Cortical Thickness in bvFTD-LC [file jamanetwopen-e211290-s001.pdf]

Supplementary Online Content

Illán-Gala I, Falgàs N, Friedberg A, et al. Diagnostic utility of measuring cerebral atrophy in the behavioral variant of frontotemporal dementia and association with clinical deterioration. *JAMA Netw Open*. 2021;4(3):e211290. doi:10.1001/jamanetworkopen.2021.1290

eTable 1. Clinical Characteristics of Participants Classified in bvFTD-LC Group
eTable 2. Structural T1-Weighted Image Acquisition Protocols by Center
eTable 3. Interrater Reliability Analyses of Visual Atrophy Scales
eTable 4. AUROC for the Differentiation Between bvFTD-HC and Healthy Controls
eTable 5. Diagnostic Accuracy for Each Discriminant Factor Analysis
eTable 6. Measures of Cerebral Atrophy by FTLD Subtypes
eTable 7. bvFTD Atrophy Score Cutoffs
eTable 8. Linear Mixed-Effects Models for the Estimation of CDR-SOB Change
eTable 9. Linear Mixed-Effects Models for the Estimation of MMSE Change
eFigure 1. Clinical Characteristics of bvFTD Participants
eFigure 2. Correlation Between bvFTD Atrophy Score and Cortical Thickness
eFigure 3. Diagnostic Accuracy of Measures of Atrophy
eFigure 4. Diagnostic Accuracy of Measures of Atrophy in the Subgroup of bvFTD-HC With Lower CDR-SOB
eFigure 5. Cortical Thickness in bvFTD-LC

This supplementary material has been provided by the authors to give readers additional information about their work.

© 2021 Illán-Gala I et al. *JAMA Network Open*.

eTable 1. Clinical Characteristics of Participants Classified in bvFTD-LC Group

Patient #	Age - sex	Clinical vignette	Last diagnosis	Reason for low certainty of underlying FTLD
#1	82 - m	Prominent dysexecutive syndrome at disease onset with apathy due to cognitive impairment. Behavioral changes also included (disinhibition, loss of empathy, sweet tooth, and lack of hygiene). Follow-up consistent with the diagnosis of Alzheimer's disease.	Behavioral/dysexecutive variant of Alzheimer's disease	Pathophysiological biomarkers suggestive of Alzheimer's disease. Autopsy: not available
#2	71 - w	Prominent dysexecutive syndrome at disease onset with apathy and simple repetitive behaviors. Minor hallucinations evidenced during follow-up, emergence of REM sleep behavior disorder and mild parkinsonism. Marked improvement with Rivastigmine.	Lewy body disease	Clinical follow-up not consistent with the diagnosis of bvFTD Autopsy: not available
#3	51 - m	Prominent behavioral syndrome reported by wife, but lack of clinical progression and other informants contacted during follow-up did not confirm behavioral changes reported by the wife. Stable during follow-up at the MCI stage.	Mild cognitive impairment	Absence of functional decline. Autopsy: not available
#4	64 - m	Progressive behavioral change 4 years before diagnosis (Apathy, disinhibition, simple repetitive behaviors). No functional impairment at diagnosis. Lack of functional decline (5 years of follow-up).	Psychiatric	Absence of functional decline. Autopsy: not available
#5	61 - m	Progressive cognitive (dysexecutive syndrome) and behavioral change 5 years before diagnosis (Irritability, hoarding, inflexibility, disinhibition, lack of empathy, childish behavior). No functional impairment at diagnosis. Lack of functional decline (4 years of follow-up).	Mild cognitive impairment, possible alcohol abuse	Clinical syndrome, functional decline, autopsy findings
#6	45 - m	Mild behavioral syndrome without functional impairment (disinhibition, lack of empathy, and eating changes). No functional decline during follow-up (7 years) and formal psychiatric diagnosis of personality disorder.	Psychiatric	Absence of functional decline. Autopsy: not available
#7	64 - m	Progressive behavioral change syndrome 4 years before diagnosis (Apathy, disinhibition, complex repetitive behaviors, dysexecutive neuropsychological profile). No functional impairment at diagnosis. Lack of functional decline (5 years of follow-up).	Psychiatric	Absence of functional decline. Autopsy: not available
#8	59 - m	Formal diagnosis of depression 6 years before bvFTD diagnosis. Slowly progressive behavioral changes including apathy, loss of empathy and compulsive behaviors. Mild functional impairment with prominent dysexecutive impairment.	Psychiatric	Absence of functional decline. Autopsy: not available
#9	66 - m	Prominent dysexecutive syndrome at disease onset. Behavioral changes appeared one year later (disinhibition, apathy, loss of empathy, sweet tooth). Also, gait changes and urinary	bvFTD	Functional decline ascertained during follow-up. Autopsy: AD

© 2021 Illán-Gala I et al. *JAMA Network Open*.

		incontinence. Ventriculoperitoneal shunt without sustained improvement.		
#10	73 - w	Formal diagnosis of depression 15 year before bvFTD diagnosis. Slowly progressive behavioral changes (two decades) at disease onset including disinhibition, loss of empathy, delusions, agitation and lack of insight. Late-onset non-convulsive seizures.	bvFTD with comorbid AD	Functional decline.<br>Autopsy: Huntington's disease
#11	66 - m	At first visit meet criteria for possible bvFTD but during follow-up the diagnosis was ruled out due to atypical features and absence of functional decline.	Psychiatric	Mild functional decline.<br>Autopsy: No evidence of neurodegeneration.
#12	57 - m	Prominent behavioral symptoms at diagnosis (apathy, loss of empathy, sweet tooth, lack of empathy, simple repetitive behaviors). Concurrent low mood, lack of hygiene. Mild functional impairment at diagnosis.	bvFTD	No clear functional decline.<br>Autopsy: no neurodegeneration
#13	54 - w	Previous history of depression and sexual abuse. Family history of psychiatric disease (bipolar disorder and schizophrenia). Prominent behavioral symptoms at diagnosis (apathy, loss of sympathy/empathy, and dietary changes) with relative sparing of cognition. Mild functional impairment at diagnosis.	Psychiatric	Absence of functional decline.<br>Autopsy: Not available
#14	47 - m	Memory complaint at diagnosis. Diagnosed of attention deficit hyperactivity disorder 10 year before. Longstanding tendency to irritability and anger. Progressive behavioral change in the last 5 years (disinhibition, apathy, mental rigidity, food preference changes with compulsive fixation on health, and increased irritability).	Psychiatric	No functional decline.<br>Autopsy: Not available
#15	55 - m	Longstanding behavioral and cognitive difficulties with recent worsening. At first visit meet criteria for possible bvFTD but during follow-up the diagnosis was ruled out (clear empathy and remorse for any poor behavior or judgment).	Psychiatric	Mild functional decline.<br>Autopsy: No evidence of neurodegeneration.
#16	66 - m	Longstanding behavioral and cognitive difficulties with recent worsening at first visit. Meet bvFTD criteria for possible bvFTD at first visit. Family history of psychiatric disorders. Diagnosis of primary psychiatric disorder during follow-up.	Psychiatric	Mild functional decline.<br>Autopsy: No evidence of neurodegeneration.
#17	65 - m	Longstanding history of episodic mood disorder (possible undiagnosed bipolar disorder). At diagnosis poor financial judgment, impulsivity, hypersexuality, and apathy were reported. Progressive cognitive and functional decline. The diagnosis of bvFTD was ruled out during follow-up.	Psychiatric	Absence of functional decline.<br>Autopsy: Not available
#18	63 - m	At diagnosis his previous personality was described as "eccentric, child-like behavior at baseline and lifelong inability to hold a job due to inappropriate behavior". Five years before referral progressive behavioral change consistent with the diagnosis of possible bvFTD	Psychiatric	Absence of functional decline.<br>Autopsy: Not available

© 2021 Illán-Gala I et al. *JAMA Network Open*.

		(disinhibition, lack of empathy, compulsive behavior, simple and complex repetitive behavior and prominent executive dysfunction). No functional decline during follow-up.		
#19	75 - m	Progressive personality change starting three years before consultation (Apathy, disinhibition, eating changes and prominent executive dysfunction). However, some atypical features were noted (alternating “high” and “low” days) without other features suggestive of Lewy body disease. No progressive functional decline.	Psychiatric	Absence of functional decline.<br>Autopsy: Not available

FTLD-related mutations (including *C9orf72*) were excluded in all cases included in bvFTD-LC group.

Abbreviations: bvFTD-LC=bvFTD with low confidence of FTLD; m=man; w=woman.

© 2021 Illán-Gala I et al. *JAMA Network Open*.

eTable 2. Structural T1-Weighted Image Acquisition Protocols by Center

	HSP I	HSP II	HCB	UCSF I	UCSF II	UCSF III	UCSF IV
Manufacturer (system)	Philips (Achieva)	Philips (Achieva)	Siemens (Tim Trio)	Siemens (Tim Trio)	Siemens (Magnetom Prisma)	Siemens (Magnetom VISION)	Siemens (Bruker MedSpec)
Magnet strength	3T	3T	3T	3T	3T	1.5T	4T
Repetition time (ms)	8.1	6.74	2300	2300	2500	5000	2300
Echo time (ms)	3.7	3.14	2.98	2.98	2.82	20	3
Slice Thickness (mm)	1	1. 2	1	1	1	1.5	1
Voxel size (mm)	0.94 x 0.94 x 1	0.9 x 0.9 x 1.2	1 x 1 x 1	1 x 1 x 1	1 x 1 x 1	1.5 x 1.5 x 1.5	1 x 1 x 1
Number of participants scanned, n (%)	80 (17%)	16 (4%)	36 (8%)	127 (28%)	126 (27%)	48 (10%)	27 (6%)
Image quality rating,[a] %	85 (3)	85 (3)	83 (2)	84 (2)	84 (2)	72 (5)[b]	85 (1)

a: Image quality rating from CAT12 software. Percentages closer to 100% indicating better quality of MRI. b: *P*<.05 compared to all other groups.

Abbreviations: HSP = hospital de sant Pau; HCB = hospital clínic de Barcelona; mm = mulimeters; ms = miliseconds.

© 2021 Illán-Gala I et al. *JAMA Network Open*.

eTable 3. Interrater Reliability Analyses of Visual Atrophy Scales

Measurement	ICC (Single measures) (n=40 measurements, 6 raters)	ICC (Average measures) (n=40 measurements, 6 raters)
Orbitofrontal	.79 (.70 to .87)	.96 (.93 to .98)
Anterior cingulate	.78 (.69 to .86)	.96 (.93 to .97)
Anterior temporal	.52 (.39 to .66)	.87 (.79 to .92)
Medial Temporal Lobe	.72 (.61 to .82)	.94 (.91 to .96)
Frontal insula	.77 (.68 to .86)	.95 (.93 to .97)
Posterior atrophy	.50 (.36 to .64)	.86 (.77 to .92)

Interrater reliability analyses of the visual atrophy scales in the training set 20 participants (40 measurements). Single measure and average measure intraclass correlation coefficients (ICC) results for each scale are shown. The 95% confidence intervals (obtained after bootstrapping of 1000 samples) are presented between parentheses. All results were statistically significant ($P<.001$).

© 2021 Illán-Gala I et al. *JAMA Network Open*.

eTable 4. AUROC for the Differentiation Between bvFTD-HC and Healthy Controls

Measure	Left hemisphere	Right hemisphere	Combined right and left hemisphere
Visual atrophy scales			
Orbitofrontal scale	.800	.804	.828
Anterior cingulate scale	.845	.839	.872
Anterior temporal scale	.780	.744	.805
Middle temporal scale	.829	.809	.845
Frontal insula scale	.801	.804	.842
Posterior atrophy scale	.533	.556	.563
Cortical thickness (Desikan atlas)			
Banks of superior temporal sulcus	.580	.644	.630
Caudal anterior cingulate	.722	.686	.764
Caudal middle frontal	.887	.867	.886
Cuneus	.647	.645	.654
Entorhinal	.845	.859	.873
Fusiform	.723	.786	.771
Inferior parietal	.704	.698	.721
Inferior temporal	.771	.800	.801
Isthmus cingulate	.661	.672	.683
Lateral occipital	.606	.631	.626
Lateral orbitofrontal	.868	.817	.851
Lingual	.587	.579	.589
Medial orbitofrontal	.758	.805	.812
Middle temporal	.838	.838	.854
Parahippocampal	.642	.617	.648
Paracentral	.676	.677	.683
Pars opercularis	.890	.879	.895
Pars orbitalis	.849	.840	.861

© 2021 Illán-Gala I et al. *JAMA Network Open*.

Pars triangularis	.875	.870	.881
Pericalcarine	.609	.538	.579
Postcentral	.672	.689	.687
Posterior cingulate	.748	.722	.767
Precentral	.739	.738	.746
Precuneus	.683	.676	.690
Rostral anterior cingulate	.761	.686	.743
Rostral middle frontal	.888	.869	.886
Superior frontal	.919	.913	.925
Superior parietal	.678	.676	.689
Superior temporal	.781	.819	.812
Supramarginal	.769	.723	.762
Frontal pole	.817	.809	.837
Temporal pole	.846	.842	.863
Transverse temporal	.683	.700	.705
Insula	.770	.774	.791
Grey matter volumes (Neuromorphometrics atlas)			
Accumbens nucleus	.864	.857	.897
Amygdala	.845	.855	.887
Caudate	.855	.855	.882
Hippocampus	.854	.873	.892
Pallidum	.641	.667	.667
Putamen	.803	.825	.859
Thalamus	.843	.845	.867

This table shows the AUROC for the differentiation between bvFTD-HC and healthy controls.

Abbreviations: bvFTD-HC=bvFTD with high confidence of FTLD; TIV=total intracranial volume.

© 2021 Illán-Gala I et al. *JAMA Network Open*.

eTable 5. Diagnostic Accuracy for Each Discriminant Factor Analysis

Measures of atrophy	bvFTD-HC vs Healthy controls	bvFTD-HC vs bvFTD-LC	bvFTD-IC + bvFTD-HC vs Healthy controls	bvFTD-IC + bvFTD-HC vs Healthy controls + bvFTD-LC
Visual atrophy scales	86%	76%	82%	83%
Cortical thickness	90%	79%	84%	84%
Subcortical volumes	88%	71%	84%	83%
Cortical thickness and subcortical volumes	91%	76%	89%	86%

For set of measures of atrophy and subsample, percentages of correct classification obtained with leave-one-out cross-validation are shown.

Abbreviations: bvFTD=behavioral variant of frontotemporal dementia, bvFTD-IC=bvFTD with intermediate confidence of FTLD, bvFTD-HC=bvFTD with high confidence of FTLD, bvFTD-LC=bvFTD with low confidence of FTLD; CI=confidence interval.

© 2021 Illán-Gala I et al. *JAMA Network Open*.

eTable 6. Measures of Cerebral Atrophy by FTLD Subtypes

	PSP	CBD	Pick's disease	Other FTLD-Tau	TDP-A	TDP-B	TDP-C	Other FTLD-TDP	FTLD-FET
n	2	5	6	6	8	8	2	23	8
bvFTD atrophy score	17 (11)	14 (6)	20 (4)	22 (6)	18 (4)	18 (8)	20 (11)	16 (5)	20 (7)
MRPI	14.5 (1.4)	13.5 (0.9)	10.5 (2.0)	11.2 (2.9)	11.0 (3.1)	11.5 (3.3)	10.5 (0.9)	11.4 (2.5)	11.4 (2.6)
Frontotemporal cortical thickness	2.3 (0.5)	2.7 (0.3)	2.2 (0.3)	2.5 (0.2)	2.4 (0.1)	2.6 (0.2)	2.4 (0.2)	2.5 (0.2)	2.4 (0.2)
Subcortical grey matter volume	1.0 (0.4)	1.2 (0.2)	0.9 (0.3)	1.2 (0.2)	1.0 (0.2)	1.2 (0.2)	1.0 (0.1)	1.2 (0.2)	1.1 (0.3)

Abbreviations: PSP=progressive supranuclear palsy; CBD=corticobasal degeneration; FTLD=frontotemporal lobar degeneration; TDP= TAR DNA-binding protein 43; FET= fused in sarcoma protein, Ewing sarcoma protein and TATA-binding protein-associated factor 15.

© 2021 Illán-Gala I et al. *JAMA Network Open*.

eTable 7. bvFTD Atrophy Score Cutoffs

bvFTD atrophy score cut-off	Sensitivity (95% CI)	Specificity (95% CI)	Likelihood ratio
Sensitive cut-off > 7	.903 (.856 to .936)	.701 (.641 to .755)	3.018
Optimal cut-off (best Youden Index) > 9	.833 (.778 to .877)	.828 (.776 to .870)	4.841
Specific cut-off > 11	.741 (.679 to .795)	.914 (.872 to .943)	.657

Proposed cut-off were calculated for the differentiation between bvFTD participants with high or intermediate confidence FTLD, and healthy controls or bvFTD participants with low confidence of FTLD (bvFTD-LC).

Abbreviations: bvFTD=behavioral variant of frontotemporal dementia; CI=confidence interval.

eTable 8. Linear Mixed-Effects Models for the Estimation of CDR-SOB Change

**Parameter**	**Atrophy measures[a]**			
	**bvFTD atrophy score**	**MRPI**	**Frontotemporal cortical thickness**	**Subcortical grey matter volume**
Intercept	6.86 [3.71 to 10.00], ***P*<.001**	5.63 [2.17 to 9.19], ***P*=.002**	5.63 [2.56 to 8.71], ***P*=.001**	5.14 [1.89 to 8.38], ***P*=.002**
Age	-.01 [-.06 to 0.3], *P*=.588	-.01 [-.06 to .04], *P*=.798	.01 [-.03 to .06], *P*=.590	.00 [-04 to .05], *P*=.910
Sex	-.35 [-1.33 to .63], *P*=.485	.64 [-1.64 to .29], *P*=.173	-.67 [-1.68 to .27], *P*=.155	.24 [-.76 to 1.23], *P*=.638
FTLD genetic mutation	.97 [-16 to 2.11], *P*=.092	1.42 [1.16 to 2.68], ***P*=.028**	1.16 [.06 to 2.26], ***P*=.039**	.49 [-.70 to 1.68], *P*=.417
Time	1.71 [0.84 to 2.59], ***P*<.001**	2.52 [1.64 to 3.40], ***P*<.001**	1.95 [.93 to 2.97], ***P*<.001**	1.90 [.80 to 3.00], ***P*=.001**
Atrophy measures[a]	1.28 [.76 to 1.81], ***P*<.001**	.61 [.07 to 1.16], ***P*=.028**	1.55 [1.01 to 2.08], ***P*<.001**	1.46 [.87 to 2.05], ***P*<.001**
Time * atrophy measure interaction	1.86 [.99 to 2.73], ***P*<.001**	-.08 [-1.11 to .95], *P*=.877	1.51 [.51 to 2.51], ***P*=.003**	1.52 [.45 to 2.58], ***P*=.006**

The estimates were obtained in linear mixed-effects models. 95% confidence intervals are shown between brackets.

a: The different atrophy measures were standardized to allow comparisons.

Linear mixed-effects models for the prediction of longitudinal change in CDRsb based on baseline characteristics (age, sex, the detection of a FTLD-related mutation, time and measures of cerebral atrophy).

Abbreviations: bvFTD = behavioral variant of frontotemporal dementia; FTLD = frontotemporal lobar degeneration; MRPI = magnetic resonance parkinsonism index.

© 2021 Illán-Gala I et al. *JAMA Network Open*.

eTable 9. Linear Mixed-Effects Models for the Estimation of MMSE Change

Parameter	Atrophy measures[a]			
	**bvFTD atrophy score**	**MRPI**	**Frontotemporal cortical thickness**	**Subcortical grey matter volume**
Intercept	23.16 [17.23 to 29.08], ***P*<.001**	24.79 [18.38 to 31.20], ***P*<.001**	24.71 [18.75 to 30.67], ***P*<.001**	25.35 [19.38 to 31.32], ***P*<.001**
Age	.06 [-.03 to 0.14], *P*=.185	.04 [-.05 to .14], *P*=.358	.02 [-.06 to .10], *P*=.614	.04 [-04 to .12], *P*=.360
Sex	-1.55 [-3.44 to .33], *P*=.106	-3.00 [-5.03 to -.98], ***P*=.004**	-1.13 [-3.04 to .79], *P*=.247	-2.48 [-4.35 to -.61], ***P*=.010**
FTLD genetic mutation	-.66 [-2.81 to 1.50], *P*=.547	-1.29 [-3.59 to 1.01], *P*=.268	-.71 [-2.88 to 1.45], *P*=.325	.31 [-1.89 to 2.52], *P*=.781
Time	-2.22 [-3.69 to -.75], ***P*=.003**	-2.80 [-4.30 to -1.30], ***P*<.001**	-2.16 [-3.87 to -.45], ***P*=.014**	-2.13 [-4.00 to -.26], ***P*=.026**
Atrophy measures[a]	-2.42 [-3.37 to -1.48], ***P*<.001**	-.87 [-1.86 to .11], *P*=.082	-2.46 [-3.39 to -1.53], ***P*<.001**	-2.69 [-3.70 to -1.68], ***P*<.001**
Time * atrophy measure interaction	-2.46 [-4.08 to -.84], ***P*<.001**	-.33 [-1.65 to .98], *P*=.614	-2.73 [-4.68 to -.78], ***P*=.006**	-2.25 [-4.45 to -.06], ***P*=.044**

The estimates were obtained in linear mixed-effects models. 95% confidence intervals are shown between brackets.
a: The different atrophy measures were standardized to allow comparisons.
Linear mixed-effects models for the prediction of longitudinal change in CDRsb based on baseline characteristics (age, sex, the detection of a FTLD-related mutation, time and measures of cerebral atrophy).
Abbreviations: bvFTD = behavioral variant of frontotemporal dementia; FTLD = frontotemporal lobar degeneration; MRPI = magnetic resonance parkinsonism index; MMSE = mini mental state examination

© 2021 Illán-Gala I et al. *JAMA Network Open*.

eFigure 1. Clinical Characteristics of bvFTD Participants

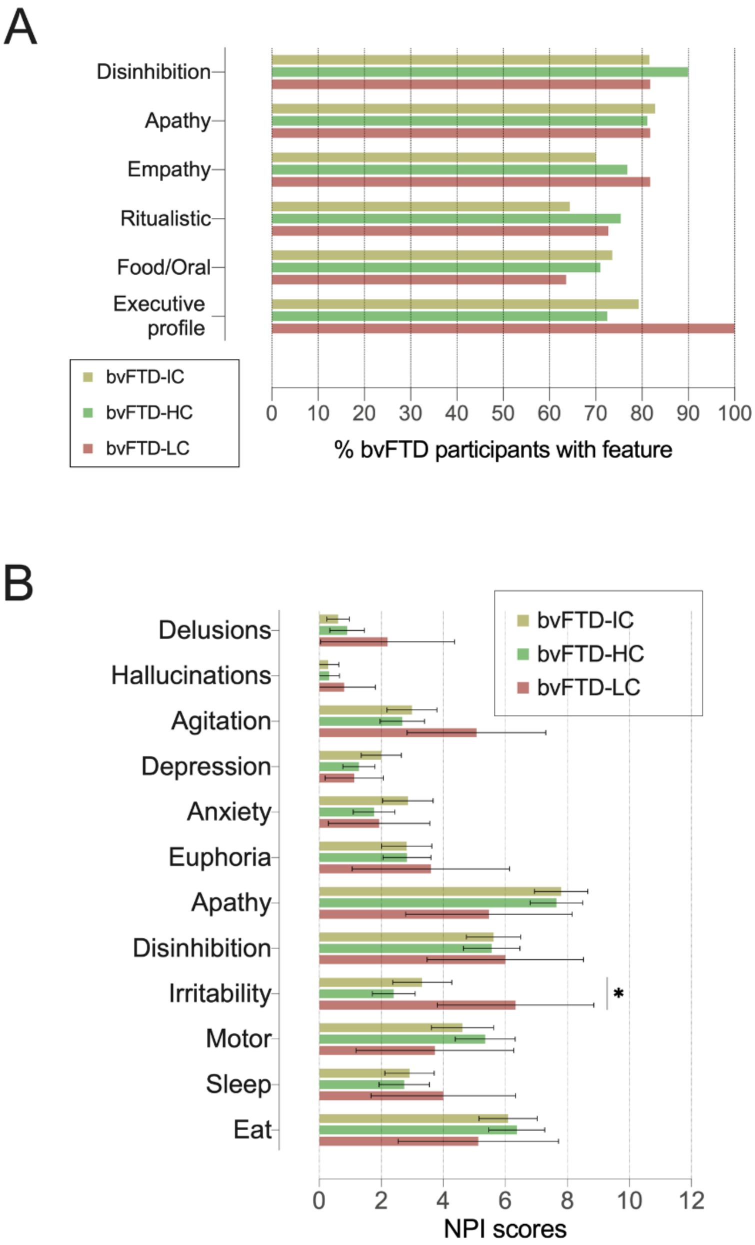

A, Behavioral profile in bvFTD-IC, bvFTD-HC and bvFTD-LC. **B,** Neuropsychiatric inventory scores in bvFTD-IC, bvFTD-HC and bvFTD-LC.

Abbreviations: bvFTD=behavioral variant of frontotemporal dementia; bvFTD-IC=bvFTD with intermediate confidence of FTLD; bvFTD-HC=bvFTD with increased confidence of FTLD; bvFTD-LC=bvFTD with low confidence of FTLD

© 2021 Illán-Gala I et al. *JAMA Network Open*.

eFigure 2. Correlation Between bvFTD Atrophy Score and Cortical Thickness

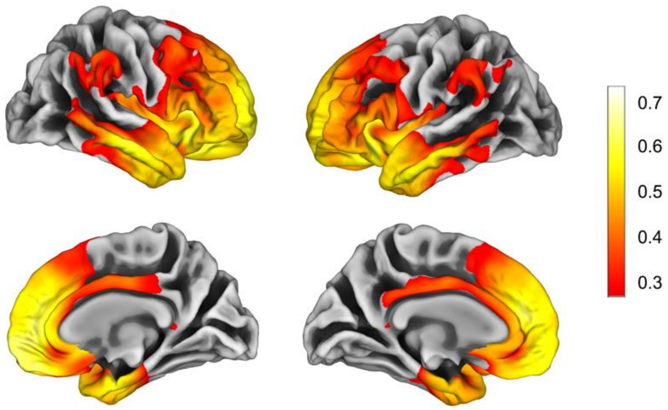

The legend represents the correlation coefficient between the bvFTD atrophy score and cortical thickness in bvFTD participants.

Abbreviations: bvFTD=behavioral variant of frontotemporal dementia.

© 2021 Illán-Gala I et al. *JAMA Network Open*.

eFigure 3. Diagnostic Accuracy of Measures of Atrophy

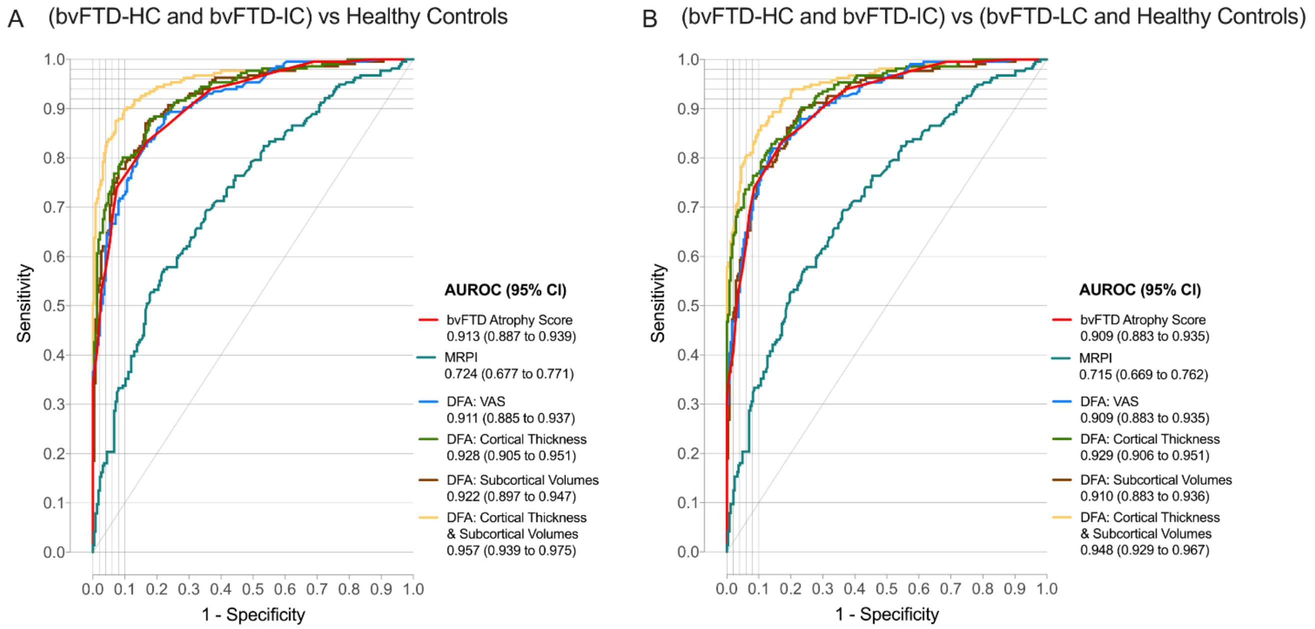

A) AUROC curves for the differentiation between bvFTD (bvFTD-HC and bvFTD-IC) and healthy controls; **B)** AUROC curves for the differentiation between bvFTD (bvFTD-HC and bvFTD-IC) and bvFTD-LC or healthy controls.

Abbreviations: AUROC=Area under the receiver operating characteristic curve; bvFTD-IC=bvFTD with intermediate confidence of FTLD, bvFTD-HC=bvFTD with high confidence of FTLD, bvFTD-LC=bvFTD with low confidence of FTLD; DFA=binary logistic regression model; CI=Confidence interval.

© 2021 Illán-Gala I et al. *JAMA Network Open*.

eFigure 4. Diagnostic Accuracy of Measures of Atrophy in the Subgroup of bvFTD-HC With Lower CDR-SOB

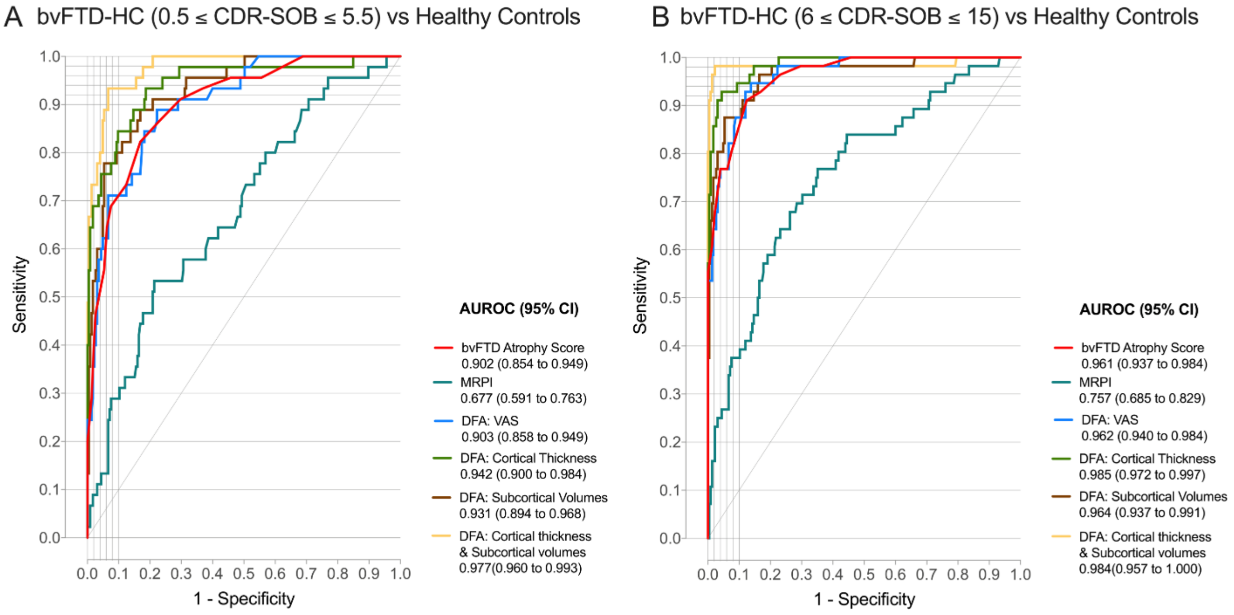

A) AUROC curves for the differentiation between bvFTD-HC with low disease severity (CDR-SOB below the median of bvFTD participants, n=45) and healthy controls; **B)** AUROC curves for the differentiation between bvFTD-HC with high disease severity (CDR-SOB above the median of bvFTD participants, n=56) and healthy controls.

Abbreviations: AUROC=Area under the receiver operating characteristic curve; bvFTD-IC=bvFTD with intermediate confidence of FTLD, bvFTD-HC=bvFTD with high confidence of FTLD, bvFTD-LC=bvFTD with low confidence of FTLD; DFA=binary logistic regression model; CI=Confidence interval.

© 2021 Illán-Gala I et al. *JAMA Network Open*.

eFigure 5. Cortical Thickness in bvFTD-LC

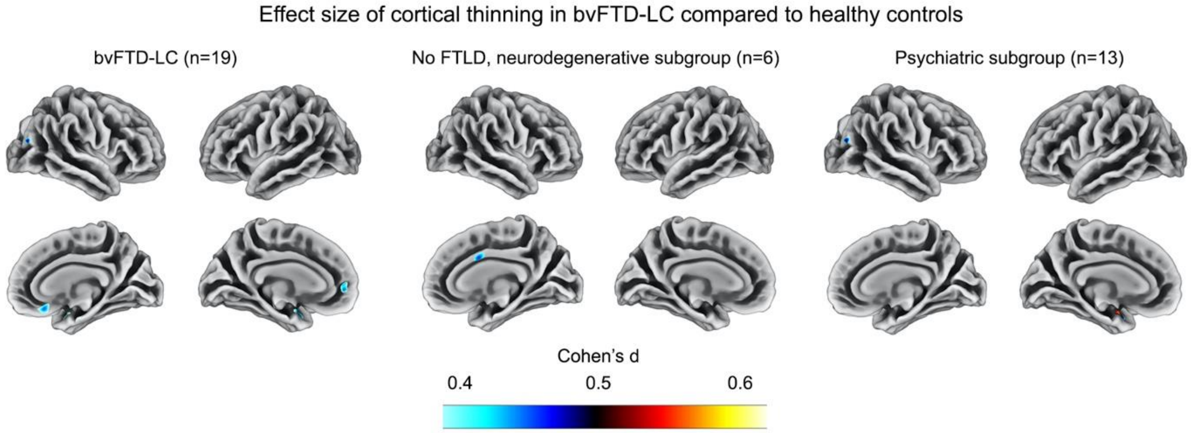

Group comparison of cortical thickness between bvFTD-noFTLD participants and healthy controls. Effect sizes are shown for regions with *P*<0.001, uncorrected. All linear mixed-effects models adjusted for age, sex, basal disease severity, and genetic status. Error bars represent 95% confidence intervals.

Abbreviations: bvFTD=behavioral variant of frontotemporal dementia; bvFTD-unk= bvFTD with unknown pathology; bvFTD-FTLD=bvFTD with FTLD; bvFTLD-noFTLD=bvFTD without FTLD.

© 2021 Illán-Gala I et al. *JAMA Network Open*.
